# Neglected rupture of the quadriceps tendon in a patient with chronic renal failure (case report and review of the literature)

**DOI:** 10.11604/pamj.2014.18.55.2664

**Published:** 2014-05-15

**Authors:** Zouhir Ameziane Hassani, Moncef Boufettal, Moustapha Mahfoud, Moradh Elyaacoubi

**Affiliations:** 1 Service of trauma and orthopedics Avicenne Hospital Chu Ibn Sina Rabat Morocco

**Keywords:** Quadriceps tendon, spontaneous rupture, renal failure, lengthening plasty

## Abstract

Spontaneous ruptures of the quadriceps tendon are infrequent injuries, it is seen primarily in patients with predisposing diseases such as gout, rheumatoid arthritis and chronic renal failure. A 32-year-old man had a history of end stage renal disease and received regular hemodialysis treatment for more than 5 years. He was admitted in our service for total functional impotence of the right lower limb with knee pain after a common fall two months ago. The radiogram showed a ‘'patella baja” with suprapatellar calcifications. The ultrasound and MRI showed an aspect of rupture of the quadriceps tendon in its proximal end with retraction of 3 cm. Quadriceps tendon repair was performed with a lengthening plasty, and the result was satisfactory after a serial rehabilitation program. The diagnosis of quadriceps tendon ruptures needs more attention in patients with predisposing diseases. They should not be unknown because the treatment of neglected lesions is more difficult. We insist on the early surgical repair associated with early rehabilitation that can guarantee recovery of good active extension.

## Introduction

Ruptures of the extensor mechanism of the knee joint are defined by the existence of a solution of continuity on the chain bone muscle tendon which provides the extension of the leg on the thighs: patellar tendon, quadriceps tendon and muscle, anterior tibial tuberosity and patella. Spontaneous ruptures of the quadriceps tendon are infrequent injuries; it is seen primarily in patients with predisposing disease such as gout, rheumatoid arthritis and chronic renal failure.

Several factors probably combine to weaken the tendon, including a loss of local vascular supply, repeated microtrauma and osteodystrophy secondary to hyperparathyroidism Through one case of spontaneous rupture of the quadriceps tendon occurred in a patient with chronic renal failure we stressed the difficulty of therapeutic management in this patient and the benefit of early rehabilitation.

## Patient and observation

Our patient, a 32 year old man, employee, without any sport practice, had a chronic renal failure, with hemodialysis dependence for 5 years, was admitted in our service for total functional impotence of the right lower limb with knee pain after a common fall two months ago.

**Figure 1:**
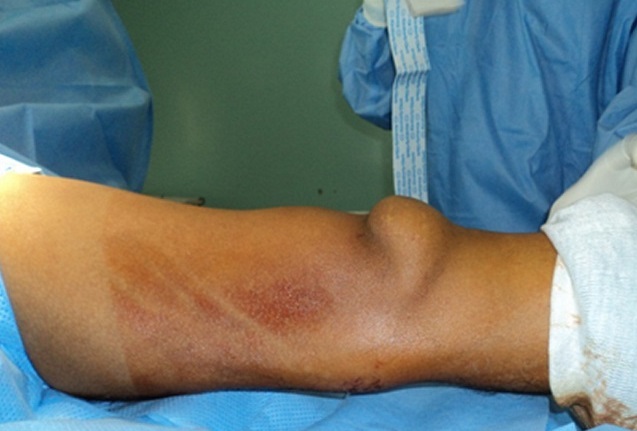
Suprapatellar defect on the right knee

**Figure 2:**
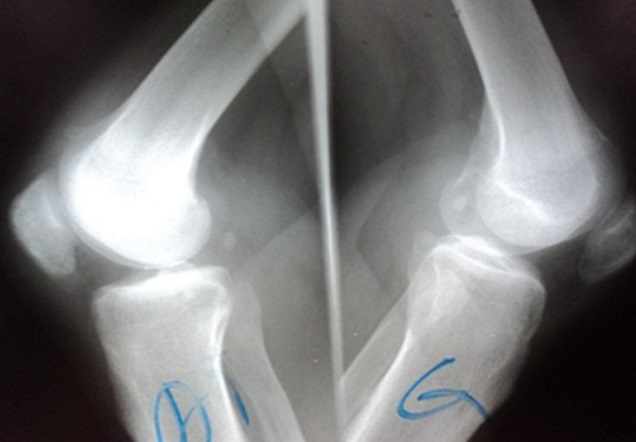
Lateral radiographs showing suprapatellar calcifications

**Figure 3:**
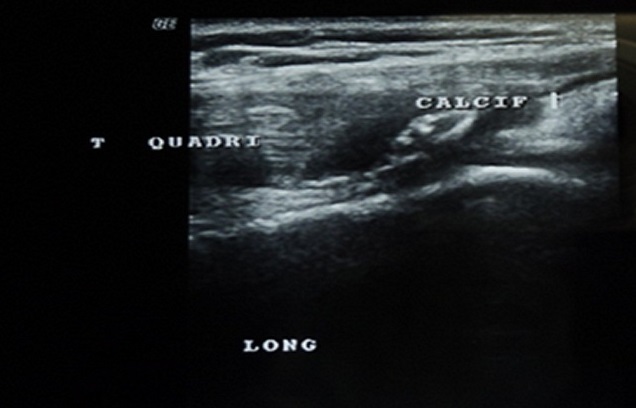
Ultrasound showing rupture of the quadriceps tendon with calcifications

Clinical examination revealed an inability to walk, a loss of active extension of the knee joint and palpable tendinous suprapatellar defect (Figure 1). Radiographic investigations showed a distally displaced patella “patella baja”with suprapatellar calcifications (Figure 2). The ultrasound showed an aspect of rupture of the quadriceps tendon in its proximal end with the presence of calcifications (Figure 3).

**Figure 4:**
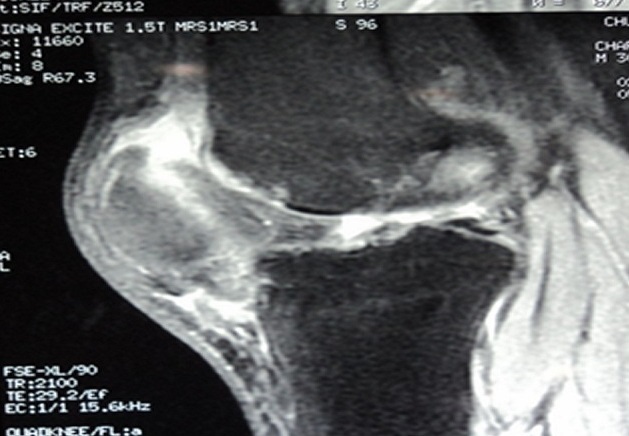
MRI confirmed the rupture of quadriceps tendon

**Figure 5:**
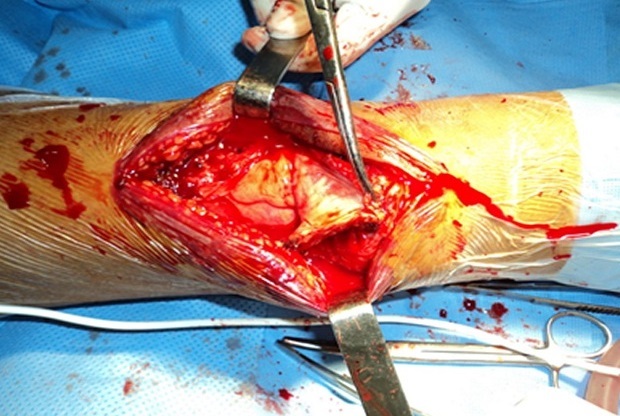
Operative view of V/Y CODIVILLA plasty

MRI confirmed the diagnosis of rupture of the quadriceps tendons and eliminates injuries of the cruciate ligament, the menisci, collateral ligaments and patellar tendon (Figure 4). Surgical exploration revealed complete rupture of the quadriceps tendon at the osteo-tendinous junction with retraction of three cm. The surgical repair consisted in tendon suture with biodegradable polyglycolic acid sutures with a lengthening plasty according to the V/Y of CODIVILLA's technique (Figure 5).

Post-operatively, the knee was immobilized with a removable knee splint for a period of six weeks. A week after, the patient was then put on program of rehabilitation based primarily on isometric contractions of the quadriceps and passive mobilization of the knee One month later, an active mobilization was initiated. The recovery of walking was allowed after 6 weeks with protection of external support (crutches). The patient was clinically evaluated each week for a month and every month.

After three years of patient follow-up, the results were graded good, based on the range of motion of the knee, the strength of the quadriceps muscle and the ability of the patient to walk with one crutch. The joint assessment found a patella mobile with active extension and flexion.

## Discussion

Quadriceps tendon ruptures are uncommon injuries. They most often occur on road accident in the population under 40 years [1]. The spontaneous rupture, secondary to a predisposing disease are not common several hypotheses are made concerning the spontaneous tendon ruptures, in the first place the vascular hypothesis where the age or diseases predisposing to rupture causing a decrease blood flow in the tendon leading to tissue degeneration which causes a spontaneous rupture in the absence of trauma [2].

Chronic renal failure can cause several complications related to the process of dialysis as amyloidosis (dialysis related amyloidosis DRA) in which there is abnormal production of beta 2 microglobulin. This molecule normally metabolized by the kidney is accumulated in chronic hemodialysis patients with rates above 30 to 40 times normal, it tends to accumulate in specific structures such as bone and tendon reducing its elasticity and predisposes to ruptures after minimal efforts [3, 4].

Besides amyloidosis, secondary hyperparathyroidism in chronic renal failure leads to dystrophic calcification and resorption of subperiostal bone causing fragility of the bone tendon junction [5–7]. This last complication is the main cause of tendon rupture.

The injury mechanism is essentially indirect muscle contraction during an extension movement of the thigh on the leg or during a forced flexion above 45° where the balance of forces between the quadriceps tendon and patellar tendon is reversed The diagnosis of tendon rupture is easy in the acute phase with the existence of a suprapatellar depression and pain on palpation a patella in the low position relative to the opposite side and inability of active extension of the knee.

In the neglected forms clinical diagnosis is more difficult, the defect may not be evident because of scar-tissue formation, and the signs are present at lower levels which give more importance to the complementary examinations.

The lateral view radiograph of the knee shows a patella baja and suprapatellar calcifications Ultrasonography of the soft tissues shows in case of total rupture, a complete interruption of tendon fibers separated by a hypoechogenous track (hematoma), but it remains operator-dependent examination [8].

In case of partial rupture it reveals a partial rupture of the tendon in the transverse plane or dissection of the fibers in the longitudinal plane.

Finally MRI shows high signal on T2 signing hemorrhage or edema. She finds its indications in the neglected forms if the clinical examination and ultrasonography cannot conclude. The treatment of a complete rupture of the quadriceps tendon is surgical but it is not always easy. The techniques are quite diverse because of the variety of lesions encountered, recent or old, and evolution of the conception of surgical techniques.

According to various authors, the repair of the neglected forms after the sixth week remains difficult because of the tendinous retraction requiring a lengthening plasty. In these cases it is recommended to use Scuderi repair and Codivilla V/Y plasty [9] and when the retraction of the quadriceps is very important it may be necessary to do a release using the technique of Judet [10]. In chronic forms, we can also see a patella baja with patellar retinaculum retraction witch must be cut on both sides of the patella during surgery [11]. Closure is done with a closed-suction drainage knee flexed for a good approximation of the wound edges.

The postoperative immobilization pedal for about 6 weeks, time of healing, is recommended this immobilisation must be made with 15° on flexion to avoid the creation or perpetuation of a patella baja.

Rehabilitation is conducted by early passive mobilization of the knee on the first day after surgery, taking into consideration the stability and solidity of surgical repair. This work will be replaced by an active rehabilitation witch it is essentially based on the stretch of the quadriceps, the gradual increase in quadriceps muscle strength and the change in execution speed of movement with the aim of this work is to reinforce the tendon.

## Conclusion

The spontaneous quadriceps tendon ruptures are uncommon. They should not be unknown because the treatment of a neglected lesion is more difficult. The large number of plasties described in the literature shows the absence of codification of this surgery. We insist on the early surgical repair associated with early rehabilitation that can guarantee recovery of good active extension.
